# Let-7a inhibits migration, invasion and tumor growth by targeting AKT2 in papillary thyroid carcinoma

**DOI:** 10.18632/oncotarget.19261

**Published:** 2017-07-15

**Authors:** Bin Zhou, Hailin Shan, Ying Su, Kai Xia, Runlong Zou, Qing Shao

**Affiliations:** ^1^ Department of Thyroid and Breast Surgery, The Affiliated Jiangyin Hospital of Southeast University Medical College, Jiangyin, China

**Keywords:** papillary thyroid carcinoma, let-7a, AKT2, tumor growth

## Abstract

Papillary thyroid carcinoma (PTC) is the most common endocrine malignancy. Increasing evidence showed that microRNAs (miRNAs) play an important role in the PTC progression. In our study, the result showed that let-7a is significantly downregulated in PTC tissues and thyroid cancer cell lines. Overexpression of let-7a suppressed PTC cell proliferation, migration and invasion. Interestingly, we found that AKT2 was a direct target of let-7a and the expression levels of AKT2 were also observed to inversely correlate with let-7a expression in PTC tissues. Furthermore, enhancing AKT2 expression partially reversed the inhibitory effects of let-7a in PTC. Taken together, these findings suggest that let-7a acts as a novel suppressor by targeting the AKT2 gene and might be a candidate target for the development of novel therapeutic strategies to treat papillary thyroid carcinoma.

## INTRODUCTION

Thyroid carcinoma is the most common endocrine malignancy of the thyroid in adults [1]. Papillary thyroid carcinoma (PTC), the most prevalent histology subtype (80% of cases), and its incidence have been increased a lot over the past few decades [2, 3]. Multifocal lesions are one of clinical characteristics of PTC and provide a power evidence of lymph node invasion and distant metastasis [4, 5]. Currently, the invasion and metastasis of PTC are the major cause of fatal outcome and, therefore, it is essential to elucidate the molecular mechanisms of PTC progression to facilitate the development of novel targeted therapies.

MicroRNAs (miRNAs) are non-protein-coding, 19-22 nucleotide RNAs that regulate gene expression post-transcriptionally by targeting their mRNAs, leading to mRNA degradation or inhibition of protein translation [6, 7]. MiRNAs include miR-187, -221, -31, -146b are known to play important roles in PTC. In particular, studies showed that expression levels of let-7a are downregulated in many types of cancers, including PTC [8–10], but whether its levels are correlated with PTC clinical stages is not known yet. In addition, the roles and mechanisms of let-7a in regulating cell proliferation, migration, invasion and tumorigenesis far from being fully elucidated.

AKT2 (v-AKT murine thymoma viral oncogene homologue 2), a key downstream effector of the phosphatidylinositol 3-kinase(PI3K) pathway [11]. Many studies show that AKT2 is involved in cancer development and play a great role in cell cycle, apoptosis, proliferation and migration [12, 13]. Recent studies indicate that AKT2 could be regulated by microRNAs. MiR-137 targeted AKT2 to suppresses tumor growth and metastasis in human hepatocellular carcinoma [14]. MiR-302b inhibited cell cycle in human hepatocellular carcinoma by targeting AKT2 [15]. The results suggest that miRNAs targeting AKT2 have an important role in carcinogenesis and are potential therapeutic agents for human cancer.

In this study, we verified that let-7a is abnormal expression in the patients and associated with cancer development. We found that let-7a overexpression significantly suppressed cell proliferation, migration and invasion *in vitro* and inhibited tumor growth *in vivo* by targeting AKT2. Our study demonstrated that let-7a negatively regulates AKT2 and is involved in PTC tumorigenesis.

## RESULTS

### Let-7a is down-regulated in papillary thyroid cancer

**Figure 1:**
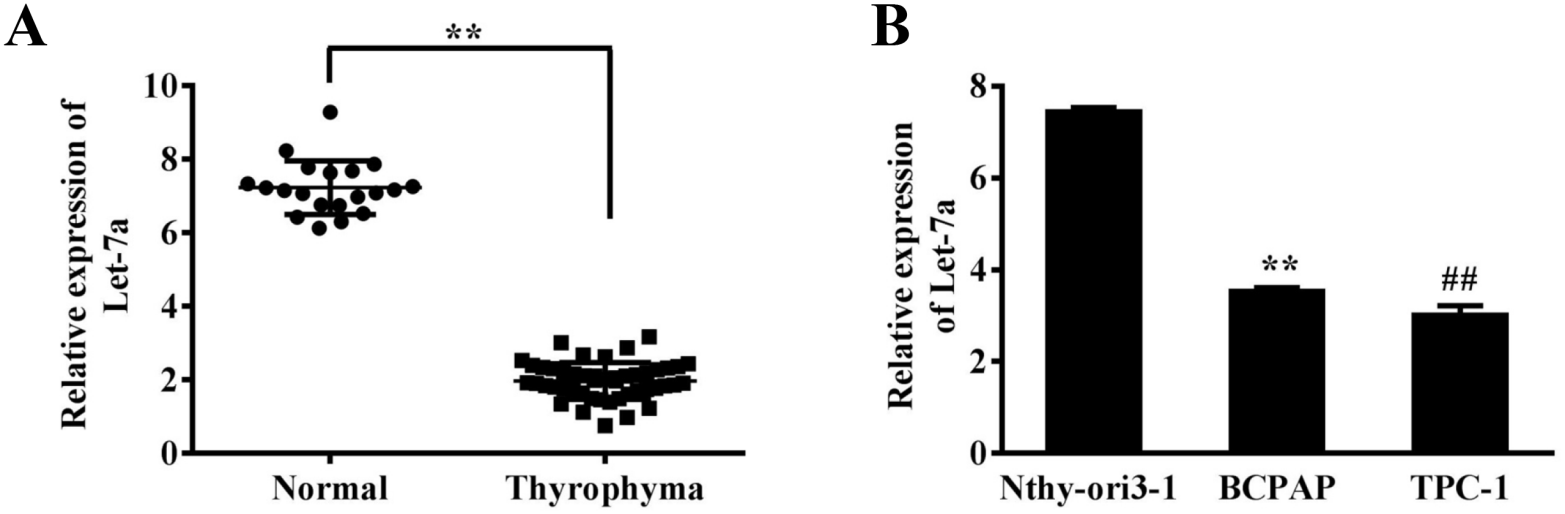
Let-7a expression is down-regulated in PTC cell lines and tissues **(A)** Relative expression levels of let-7a in 47 PTC and 21 normal thyroid tissues were analyzed by qRT-PCR. **(B)** Relative expression levels of let-7a expression in the Nthy-ori3-1, BCPAP and TPC-1 cell lines, Error bars represent the mean±SD of triplicate experiments. (**) (^##^) Significant difference when compared with the normal tissues or cells group (*P*< 0.01).

To investigate the role of let-7a in PTC, we measured expression levels of let-7a in 21 normal thyroid tissues and 47 papillary thyroid cancers. Quantitative reverse transcriptase (qRT-PCR) assay showed that let-7a expression levels were significantly decreased in PTC samples compared with the normal papillary tissues (Figure 1A). Based on the clinical information of patients, we found that the aberrant expression of let-7a was related with the pathological stages including TNM stage and lymph node metastasis in PTC patients (P<0.05), although its expression had not significant correlation with gender, age and tumor size (P>0.05) (Table 1). Moreover, the let-7a expression levels were also detected in the papillary derived cell lines BCPAP and TPC-1. The results demonstrated that the expression of let-7a was reduced compared to normal control human thyroid epithelial cells (Nthy-ori3-1) significantly (Figure 1B).

**Table 1:** Association between let-7a expression and clinicopathological features of papillary thyroid carcinoma patients

Parameters	Patients (n=47)	Expression of let-7a (Mean±SEM)	P-value
Gender:			
Male	9	2.182±0.562	0.161
Female	38	1.914±0.497	
Age (years):			
≤40	32	1.917±0.487	0.358
>40	15	2.067±0.574	
Size:			
<30 mm	28	1.924±0.541	0.509
≥30 mm	19	2.026±0.483	
Lymph node metastasis:			
Absent	25	2.142±0.533	0.010*
Present	22	1.764±0.420	
Stage:			
I–II	27	2.231	0.001*
III–IV	20	1.768	

*P < 0.05 was considered to be a statistically significant difference.

### Let-7a suppressed PTC cell proliferation, colony formation, migration and invasion

**Figure 2:**
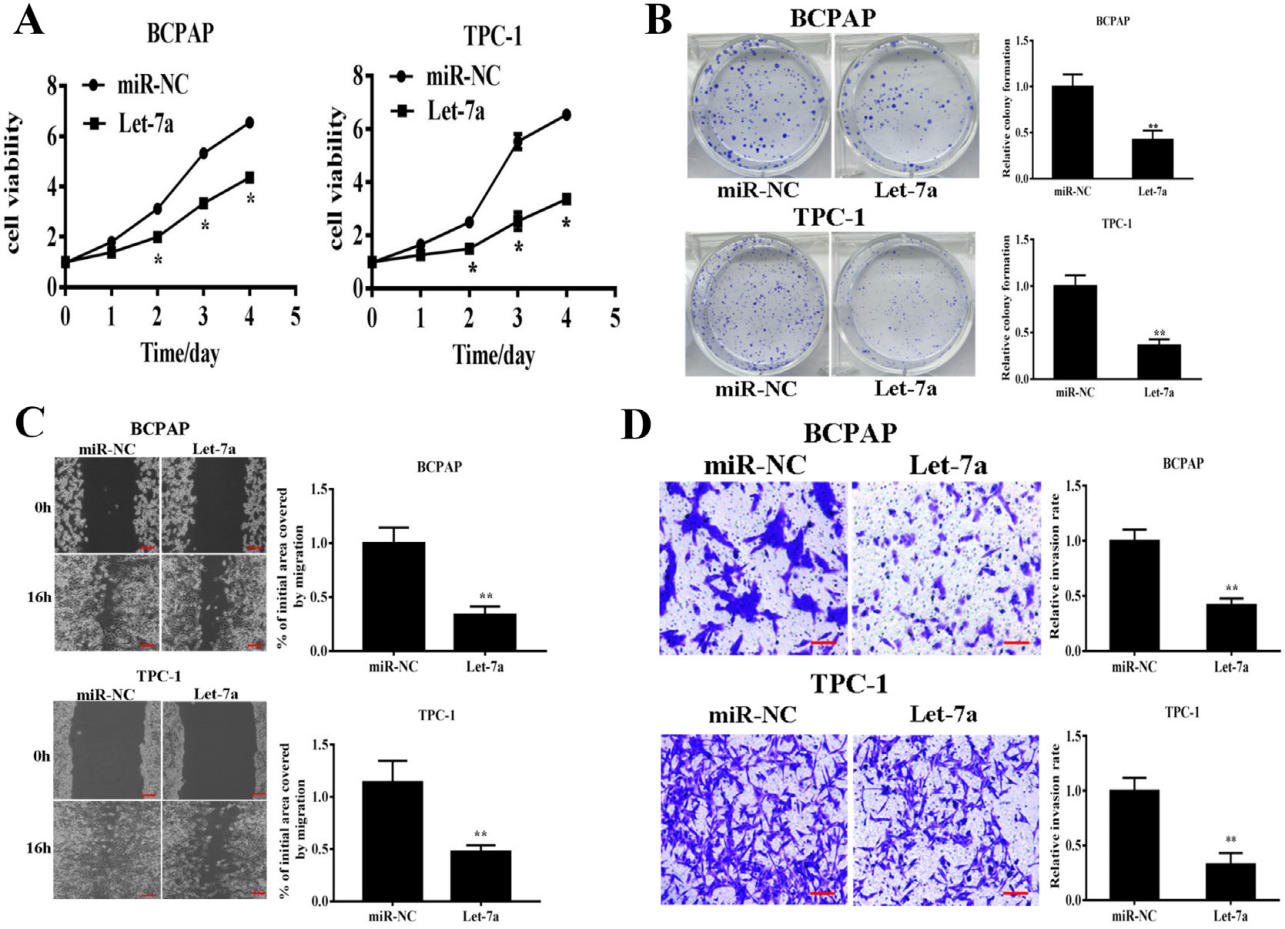
Let-7a overexpression inhibits cell proliferation, colony formation, migration and invasion BCPAP and TPC-1 cells were infected with lentivirus packing let-7a and expression. **(A)** Cell proliferation was assessed by CCK-8 kit. **(B)** Analysis of colony formation was shown. **(C)** Cells were cultured until reached 90% confluence. 20μl tips were used to scratch the cells layers to form a wound. The wound gaps were photographed and measured. **(D)** Transwell invasion assays was performed. After fixed, stained, and photographed, the cells in the bottom of the invasion chamber was measured by the absorbance at 570nm. Error bars represent the mean±SD of triplicate experiments. (**) Significant difference when compared with the miR-NC group (*P*< 0.01). Bar =200μm.

To test the direct role of let-7a in PTC cells, we established stable cell lines by infecting BCPAP and TPC-1 cells with lentivirus carrying let-7a or the negative control (miR-NC) followed by puromycin selection. Cell viability assay indicated that overexpressing let-7a significantly reduced cell growth rate 48h after seeding (Figure 2A). Consistently, overexpression of let-7a dramatically suppressed colony formation (Figure 2B). Given that PTC was easy to metastasize, we next examined whether let-7a could affect PTC cells migration and invasion. Our results showed that forced expression of let-7a markedly reduced the migration and invasion ability in PTC cells (Figure 2C–2D). Meanwhile, transfection of let-7a inhibitor (let-7a-I) into BCPAP cells dramatically induced cell proliferation, colony formation, migration and invasion (Supplementary Figure 1). Collectively, these results indicated that let-7a is associated with PTC development.

### AKT2 was a direct target of let-7a

**Figure 3:**
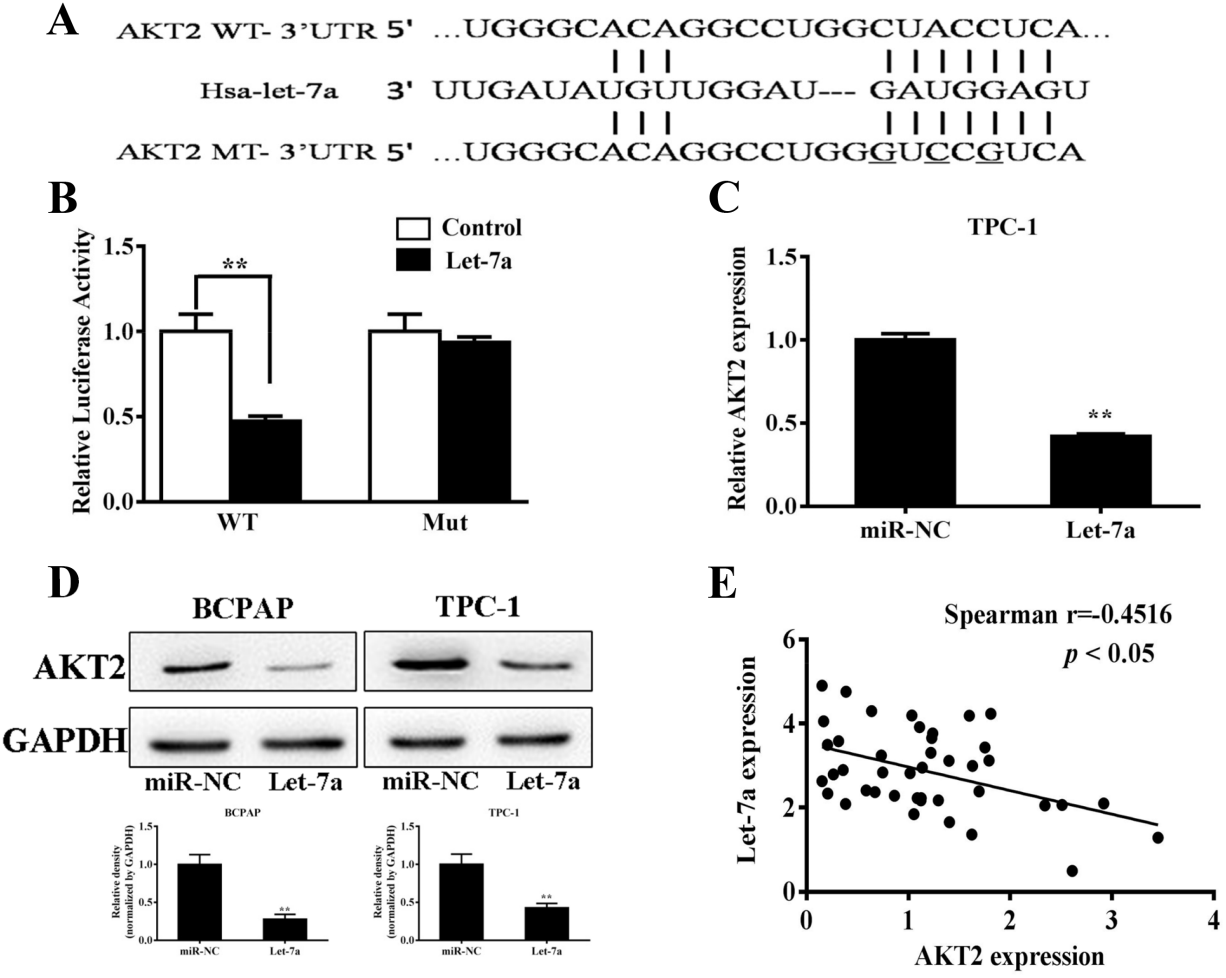
AKT2 was a direct target of let-7a **(A)** Putative seed-matching sites or mutant sites (underlined) between let-7a and 3’-UTR of AKT2. **(B)** Relative luciferase activities of AKT2 wild type (WT) and mutant (Mut) 3’- UTR were assayed and normalized to those of renilla activities (internal control). Data were presented as means±SE from three independent experiments with triple replicates per experiment. **(C)** TPC-1 cells stably expressing scrambled control (miR-NC) and let-7a were subjected to qRT-PCR analysis for AKT2 and GAPDH expression levels. **(D)** Total proteins were subjected to western blotting and detected for AKT2 expression levels. The density of AKT2 expression levels were quantified and normalized to the level of GAPDH. **(E)** Spearman’s correlation analysis was used to determine the correlation between the expression levels of AKT2 and let-7a (Spearman’s correlation analysis, r=-0.4516; *p*<0.05).

To better understand molecular action of let-7a in PTC, we searched for potential targets by TargetScan (http://www.targetscan.org), and tested the potential targets using bioinformatic database. We found that let-7a has a putative binding site to the 3’-UTR region of AKT2 with conserved sequence in human (Figure 3A). The AKT2 3’-UTR with wild type or mutant let-7a binding sites, was cloned into pMIR-REPORT miRNA reporter vector. Forced expression of let-7a markedly decreased wild type reporter activities by 47.2%, whereas no significance difference in the mutant reporter luciferase activities (Figure 3B), suggesting that AKT2 is a direct target of let-7a. To detect whether let-7a can influence the AKT2 expression in the transcriptional level, qRT-PCR was performed. The result showed that let-7a could inhibit the mRNA level of AKT2 (Figure 3C). Meanwhile western blotting showed that let-7a overexpression inhibited AKT2 protein expression (Figure 3D), while let-7a inhibition up-regulated the AKT2 protein levels compared with negative control (Supplementary Figure 2). In order to investigate the relationship between expressions of let-7a and AKT2 in human PTC specimens, we analyzed the mRNA levels of AKT2 expression in PTC specimens and normal thyroid tissues by RT-PCR. Then, we determined the correlation between AKT2 levels and let-7a levels in the same tissues. As shown in Figure 3E, Spearman’s correlation analysis demonstrated that the mRNA levels of AKT2 expression in thyroid carcinoma tissues were inversely correlated with let-7a expression levels (Spearman’s correlation r=-0.4716, *p*<0.05). Thus, lower levels of let-7a in PTC are associated with induction of AKT2, which may in turn induce tumorigenesis.

### Forced AKT2 expression partially restored the inhibitory effects of let-7a in PTC cells

**Figure 4:**
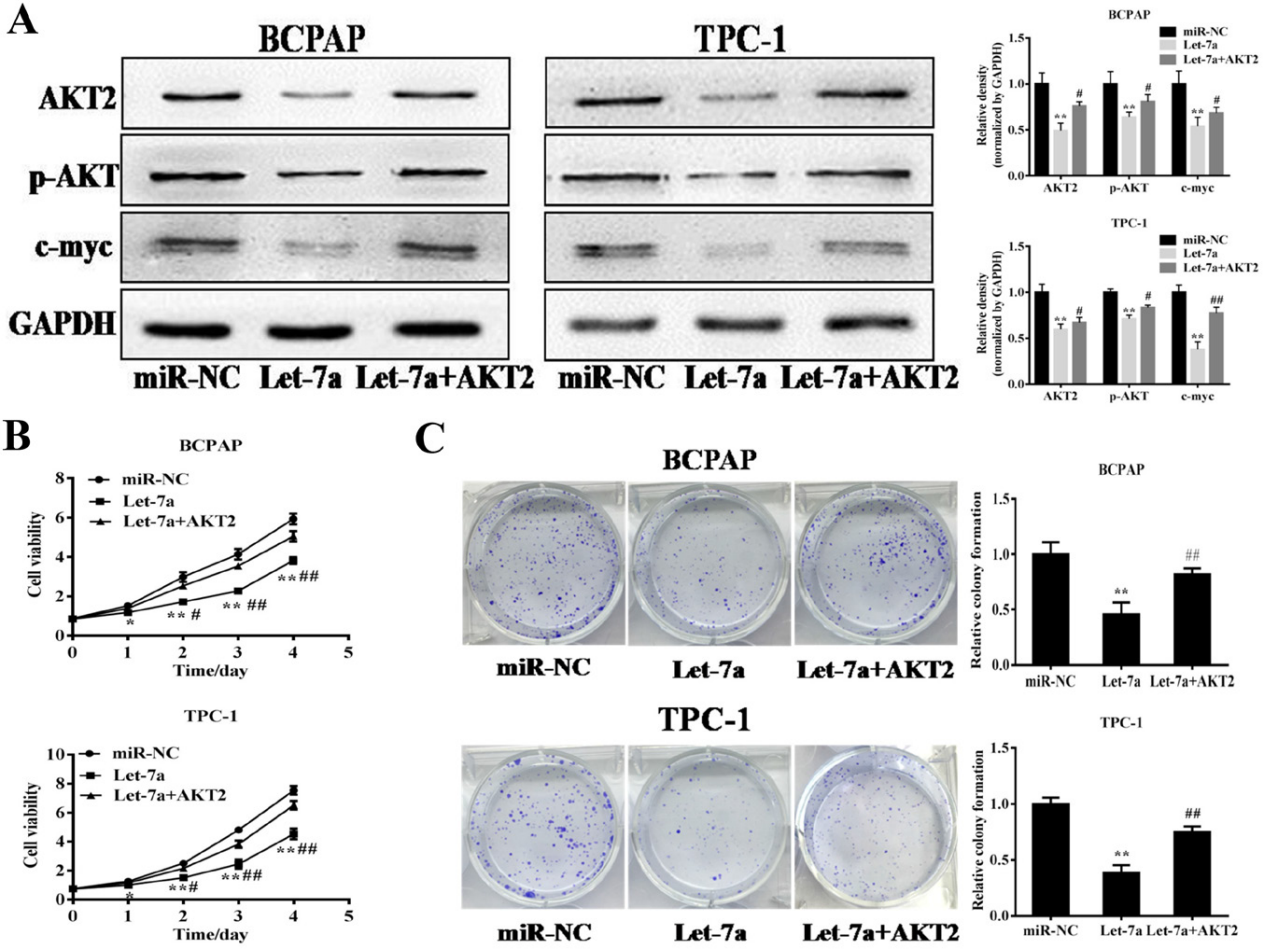

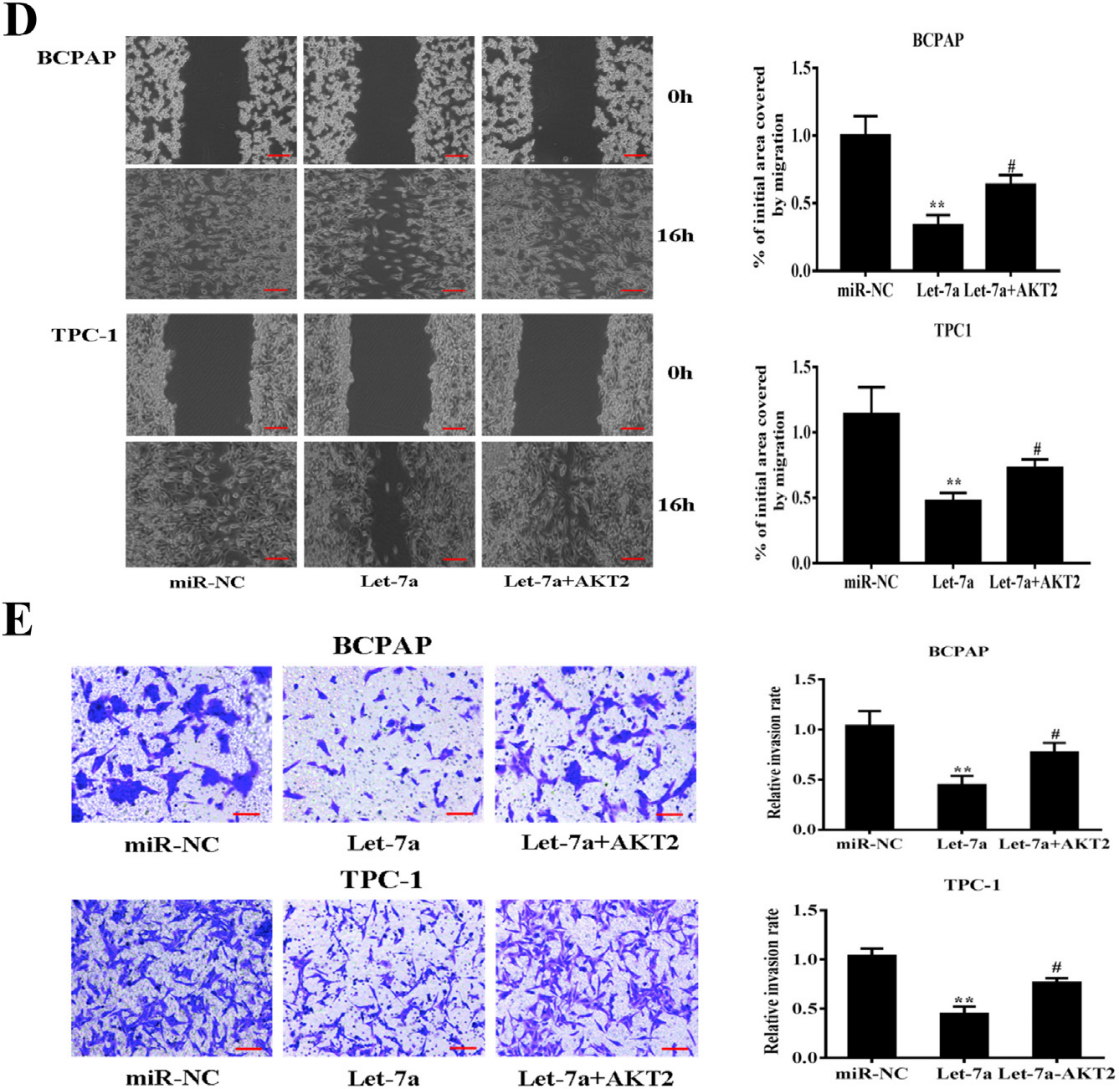
Forced expression of AKT2 reverses let-7a-suppressed cell proliferation, colony formation, migration and invasion **(A)** The stable cells of BCPAP and TPC-1 cells overexpressing let-7a were transfected with AKT2 cDNA without 3’-UTR region. After 72 h cultures, immunoblotting assay was performed as described above. The density of relative proteins expression levels were quantified and normalized to the level of GAPDH. **(B)** The Cell Counting Kit-8 (CCK-8) assay showed that BCPAP and TPC-1 cells stably expressing let-7a grew slower than cells stably expressing miR-NC. **(C, D, E)** Colony formation, migration and transwell invasion assays of BCPAP and TPC-1 cells stably expressing miR-NC or let-7a were performed. Error bars represent the mean±SD of triplicate experiments. (*, **) Significant difference when compared with the miR-NC group (*P*< 0.05)(*P*< 0.01). (^#^, ^##^) Significant difference when compared with the let-7a group (*P*< 0.05, *P*< 0.01). Bar =200μm.

Increasing studies demonstrated AKT2 had an important role in cancers as an oncogene [16–19]. Previous studies validated AKT/c-myc pathway is thought to be involved in tumorigenesis [20, 21]. To further understand the let-7a–induced anti-tumor process by targeting AKT2 in PTC cells, the stable cells of BCPAP and TPC-1 cells overexpressing let-7a were transfected with AKT2 cDNA without 3’-UTR. The western blotting results confirmed the effect of AKT2 cDNA transfection (Figure 4A). Meanwhile, the result also found that let-7a inhibited p-AKT and c-myc protein levels, while AKT2 overexpression could partially release the suppression (Figure 4A). Further, the rescue experiments showed that AKT2 significantly attenuate the inhibition of PTC cell proliferation, colony formation, migration and invasion induced by let-7a overexpression (Figure 4B–4E), suggesting that the inhibitory effect of let-7a in PTC cells is regulated by its target AKT2.

### Let-7a suppressed tumor growth by targeting AKT2 in nude mice

**Figure 5:**
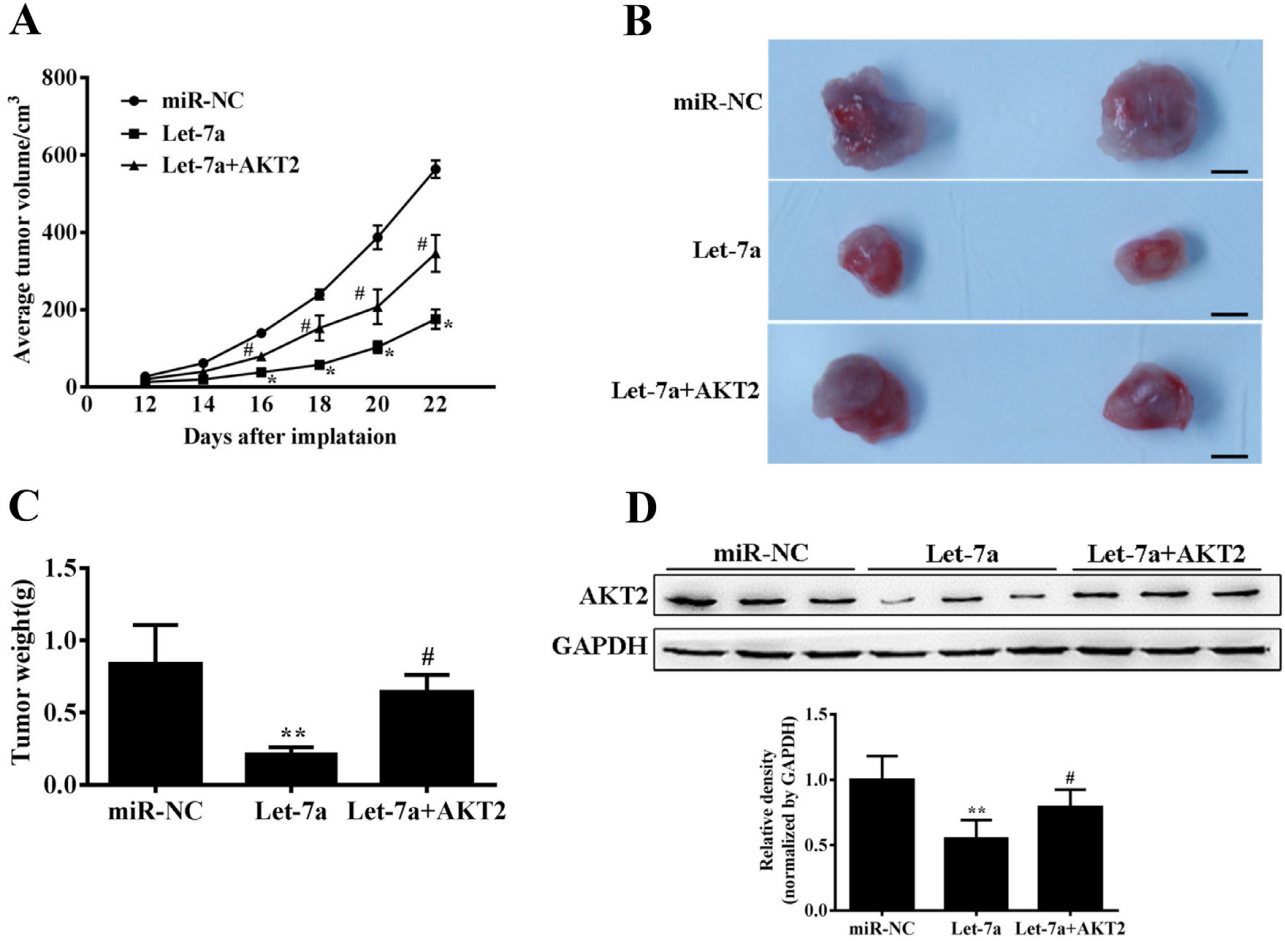
Let-7a suppresses tumor growth **(A)** Nude mice were injected subcutaneously with 2x10^6^ TPC-1 cells stably expressing let-7a, let-7a+AKT2 or miR-NC. Each treatment group contained 4 tumors. When the xenografts were visible, the width and length of tumors were measured. The tumor growth curve upon implantation was showed. **(B)** Representative pictures of xenograft tumors are shown. Bar=200μm. **(C)** Tumor growth were significantly inhibited in let-7a-overexpression group, and partly upregulated in let-7a+AKT2-overexpression group. The tumor weight was measured for each xenograft. **(D)** The levels of AKT2 from the tumor tissues of let-7a-expressing group were much lower than that of control group by immunoblotting assay, and partly upregulated in let-7a+AKT2-overexpression group. The density of AKT2 expression levels were quantified and normalized to the level of GAPDH. Error bars represent the mean±SD, (*, **) Significant difference when compared with the miR-NC group (*P*< 0.05)(*P*< 0.01). (^#^, ^##^) Significant difference when compared with the let-7a group (*P*< 0.05, *P*< 0.01). Bar =200μm.

To further investigate the role of let-7a in tumor growth *in vivo*, TPC-1 cells stably expressing miR-NC, let-7a and let-7a+AKT2 were subcutaneously injected into posterior flank of nude mice. Xenograft tumor volumes were measured every 2 days when they were palpable. On day 12 after implantation, tumors from cells overexpressing let-7a were significantly smaller than those from control cells, while the suppression of tumor growth was released in let-7a+AKT2 group (Figure 5A). Nude mice were sacrificed on day 22 after implantation, and xenografts were collected and weighed. Consistent with tumor growth curve, the tumor size and weight of let-7a group were much smaller than that of the control group, and the let-7a+AKT2-overexpression group were partially reversed the inhibitory effect of let-7a (Figure 5B–5C). Furthermore, the protein levels of AKT2 in xenografts were significantly suppressed in let-7a group, and partly upregulated in let-7a+AKT2 group. (Figure 5D). These results confirmed that let-7a could inhibit tumor growth by targeting AKT2.

## DISCUSSION

Papillary thyroid carcinoma (PTC) is the most common thyroid malignancy in clinical practice. The early phase of PTC has no specfic manifestations, which makes it difficult to identify [22]. Recent study has showed that miRNAs are abnormal expression in the PTC. For example, miR-101 can suppresses migration and invasion by targeting Rac1 in PTC [23]. MiR-199a was downregulated in PTC, and its overexpression resulted in the reduction of PTC growth *in vitro* and *in vivo* [24]. Let-7a acts as a kind of new miRNAs, and has been featured by a tumor suppressor in different human tumors. Let-7a also targeted some related genes to affect signal pathways of tumors. As reported let-7ainhibited cell proliferation via targeting of E2F2 in osteosarcoma cells [25]. Let-7a has a close relationship with PKM2 signal pathway, and let-7a exerts an anti-tumor effect on different tumors [26–29]. In this study, we analyzed that the expression levels of let-7a was downregulated in human PTC tissues compared with normal tissues. Overexpression of let-7a significantly inhibited cells proliferation, migration, invasion and tumor growth, and suggested an essential role of this miRNA in mediating PTC oncogenesis and tumor behavior.

AKT2 was one of the isoform of the AKT. Previous reports suggested that AKT2 affected the development of tumors through relative signal pathways such as PI3K/AKT/c-myc pathway, which affected cell proliferation, migration and invasion of tumors, and induces cell apoptosis, and inhibited tumor growth in animal models in variety tumors. [20, 30, 31]. Interestingly, we found that AKT2 was a direct target of let-7a. Luciferase reporter assays and western blotting confirmed direct and negative regulation of AKT2 expression by let-7a through binding to the 3’-UTR region. Moreover, an inverse correlation was observed between the let-7a and AKT2 expression in PTC tissues. Proliferation, colony formation assay migration assay, and invasion assay confirmed that enhancing AKT2 expression partly rescued the inhibitory effects of let-7a in PTC cells. Animal models also demonstrated that let-7a inhibited tumor growth by targeting AKT2. Further experiments are needed to deeply elucidate how AKT2 is involved in let-7a -induced tumor suppression.

Taken together, our study provides experimental evidence that let-7a suppresses cell growth in PTC by directly targeting AKT2. Furthermore, let-7a is downregulated in PTC tissues and let-7a levels are inversely related with the protein levels of AKT2, suggesting that let-7a could be a specific biomarker of PTC. Despite having a better understanding of the molecular events that govern the PTC than ever before, it remains a clinical challenge in the treatment of PTC. Identification of new biomarkers that play a central role in the progression of glioma will benefit diagnosis and targeting therapy of the disease.

## MATERIALS AND METHODS

### Human tissue samples

Human PTC specimens (n=47) and normal adjacent tissue samples (n=21) were collected from patients undergoing standard surgical procedures in the Jiangyin Hospital. Each sample was frozen in liquid nitrogen immediately. The collection and use of patient samples was reviewed and approved by the Ethics Committee of the Jiangyin Hospital of Southeast University Medical College (Jiangyin, China), and written informed consent was obtained from each patient.

### Cell culture and antibodies

Human PTC cell lines TPC-1, BCPAP and human thyroid epithelial cells(Nthy-ori3-1) were cultured in Dulbecco’s modified Eagle’s medium(DMEM) supplemented with 10% fetal bovine serum (FBS), 100units of penicillin/mL, and 100ng of streptomycin/mL. Human embryonic kidney 293T cells were cultured in DMEM supplemented with 10% FBS, 100units of penicillin/mL, 100ng of streptomycin/mL, and 2mmol/mL glutamine. All the cells were incubated at 37°C supplemented with 5% CO_2_. Antibodies against AKT2 were purchased from Cell Signaling Technology. Antibodies against glyceraldehyde 3-phosphate dehydrogenase (GAPDH) were obtained from Bioworld.

### Lentivirus packaging and stable cell lines

The hsa-let-7a and hsa-miR-negative control (miR-NC) lentiviral constructs were purchased from Genechem (Shanghai, China). To establish stable cells expressing let-7a and miR-NC, lentiviruses were packaged using Lentiviruses Packaging System according to the manufacturer’s instruction (Genechem, Shanghai, China). The lentiviral supernatant obtained from HEK-293 cells was used to transduce BCPAP and TPC-1 cells. Stable cell lines were selected using puromycin.

### miRNA inhibitor transfections

Let-7a inhibitor (Let-7a-I) and negative control (miR-NC-I) were purchased from Genechem (Shanghai, China). Lipofectamine 2000 (Invitrogen, Carlsbad, CA, USA) was used for transfection according to the manufacturer’s instructions. All of the small RNAs were used at a final concentration of 50 nM.

### RNA extraction and quantitative real time (qRT-PCR)

Total RNA extraction was performed with TRIzol reagent (Invitrogen, Carlsbad, CA) according to the manufacturer’s instructions. To measure let-7a expression levels, RNAs were transcribed by stem-loop RT primer using PrimeScript RT Reagent Kit (Takara, Dalian, China). QRT-PCR was performed using SYBR Premix DimerEraser (Takara, Dalian, China) on a 7900HT system. U6 levels were used as an internal control, respectively.

### Cell proliferation assay

Cells in the logarithmic phase of growth were seeded at 3000 per well and cultured in 96-well plates. Cell proliferation was assayed using the Cell-Counting Kit 8 (Dojindo Laboratories, Kumamoto, Japan) according to the manufacturer’s instructions at indicated time points. Three independent experiments were performed in triplicate.

### Anchorage-independent colony formation assay

1 mL of 0.8% solidified SeaPlaque agarose (BMA, ME, USA) was added to each well of 6-well plates; 5000 cells were mixed with 1 mL of 0.4% SeaPlaque agarose and added onto the top of the well. After about 2 weeks, colonies were fixed with methanol for 15 min and stained with 0.1% crystal violet. Colonies with diameter more than 1.5 mm were counted. Experiments were performed in triplicate for three times.

### Wound healing assay

Cells were cultured in six-well plates until reached 90% confluence. 20-μL tips were used to scratch cell layers to form wounded gaps, washed with PBS twice and incubation for 16h. The wounded gaps were photographed and analyzed by measuring the distance of migrating cells in three different areas for each wound. Cells were detected for three wells per group.

### Invasion assay

Invasion assay was determined using 24-well BD Matrigel invasion chambers (BD Biosciences, Cowley, UK) in accordance with the manufacturer’s instructions. Dulbecco’s modified Eagle medium with 10% fetal bovine serum was added to the lower compartment as the chemotactic factor. 8×10^4^ cells in serum-free Dulbecco’s modified Eagle medium were added to the upper compartment of the chamber. After 24 h of incubation, the cells that had not invaded through the pores were carefully removed. The filters were then fixed in 3% paraformaldehyde, which was followed by 0.1% crystal violet staining. Then the bottom cells were counted under a light microscope in 5 random visual fields (200×). Each experiment was repeated 3 times.

### Dual-luciferase reporter assay

For dual-luciferase assay, AKT2 3’-UTR containing predicted let-7a seed-matching sites (wide type, WT) and corresponding mutant sites (mut) were amplified by PCR from cDNA of TPC-1 and inserted into pMIR-REPORTER vector (Ambion, CA, USA). Wild-type and mutant constructs were confirmed by sequencing. 293Tcells were seeded in a 24-well plate and cotransfected with either wild-type (WT) or mutant-type (mut) luciferase reporter plasmids containing AKT2-3’-UTR, pGL4.74 vector (Ambion), and equal amounts of let-7a, miR-NC (GenePharma, Shanghai, China) using Lipofectamine 2000 (Invitrogen) according to the manufacturer′s instruction. Luciferase activities were measured 24 h after transfection using a dual luciferase assay kit (Promega, WI, USA). Experiments were performed in triplicate with three independent replicates.

### Western blotting

The cells were homogenized in a chilled lysis buffer containing 10mM Tris–HCl (pH 7.4), 1%NP-40, 0.1% deoxycholic acid, 0.1%SDS, 150mM NaCl, 1mM EDTA and 1% protease inhibitors. Tumor tissues from nude mice were grinded into powder in liquid nitrogen with RIPA buffer, and the total tissue proteins were extracted as described above. Total proteins were collected by centrifugation. Twenty micrograms of lysate proteins were separated by SDS–PAGE and subsequently transferred to a nitrocellulose membrane. Membranes were blocked with 5% non-fat dry milk for 2 h and incubated with primary antibodies. Protein bands were detected by incubation with horseradish peroxidase-conjugated antibodies and visualized with an enhanced chemiluminescence reagent.

### Xenograft tumor model in nude mice

For tumor growth assay, male nude mice [BALB/cA-nu, 6-wk-old] were purchased from SLAC Animal Center and maintained in special pathogen-free (SPF) conditions. Aliquots of cells (2×10^6^) were suspended in 150μl of FBS-free DMEM medium and subcutaneously injected into posterior flank of nude mice (n=3). Tumor size was measured using vernier caliper every 2 d when they were visible, and tumor volume was calculated according to the formula: volume=0.5×length×width^2^. The mice were euthanized after 22 days, and tumors were weighed.

### Statistical analysis

The data represent mean SE except where indicated. Statistical analysis was performed based on a Student’s t-test at the significance level of *p*<0.05. Spearman’s non-parametric correlation test was performed to test the correlation between the expression levels of let-7a and AKT2.

## SUPPLEMENTARY MATERIALS FIGURES


