# Supplementary material for: Let-7a inhibits migration, invasion and tumor growth by targeting AKT2 in papillary thyroid carcinoma
Source: Oncotarget. 2017 Jul 15;8(41):69746–55. doi: 10.18632/oncotarget.19261 (PMC5642513; doi:10.18632/oncotarget.19261)
Supplement: Supplementary file 1 [file oncotarget-08-69746-s001.pdf]

Let-7a inhibits migration, invasion and tumor growth by targeting AKT2 in papillary thyroid carcinoma

SUPPLEMENTARY MATERIALS

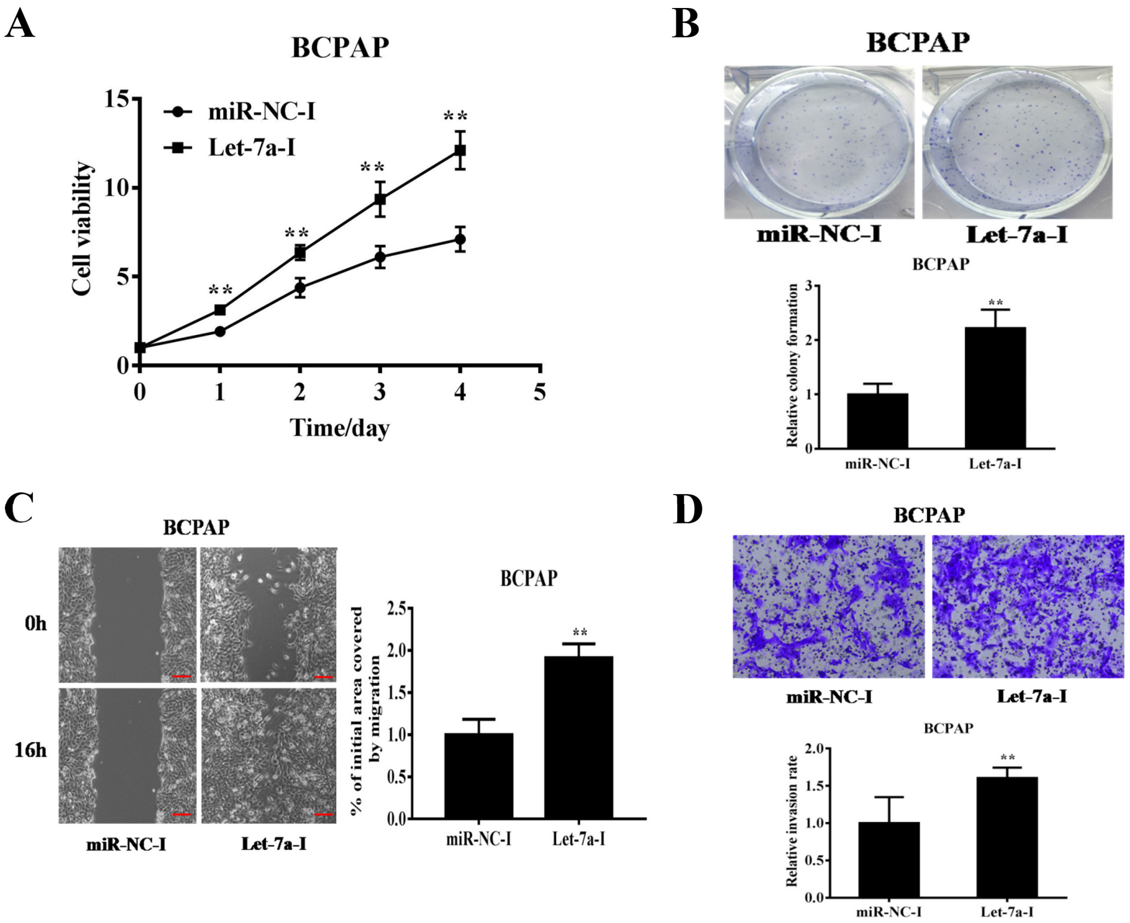

Supplementary Figure 1: Let-7a inhibitor accelerated cell proliferation, colony formation, migration and invasion in BCPAP cells. BCPAP cells were transfected with let-7a inhibitor (let-7a-I) or negative control (miR-NC-I). **(A)** Cell proliferation was assessed by CCK-8 kit. **(B)** Analysis of colony formation was shown. **(C)** Cells were cultured until reached 90% confluence. 20μl tips were used to scratch the cells layers to form a wound. The wound gaps were photographed and measured. **(D)** Transwell invasion assay was performed. After fixed, stained, and photographed, the cells in the bottom of the invasion chamber was measured by the absorbance at 570nm. Error bars represent the mean±SD of triplicate experiments. (**) Significant difference when compared with the miR-NC-I group (P< 0.01). Bar =200μm.

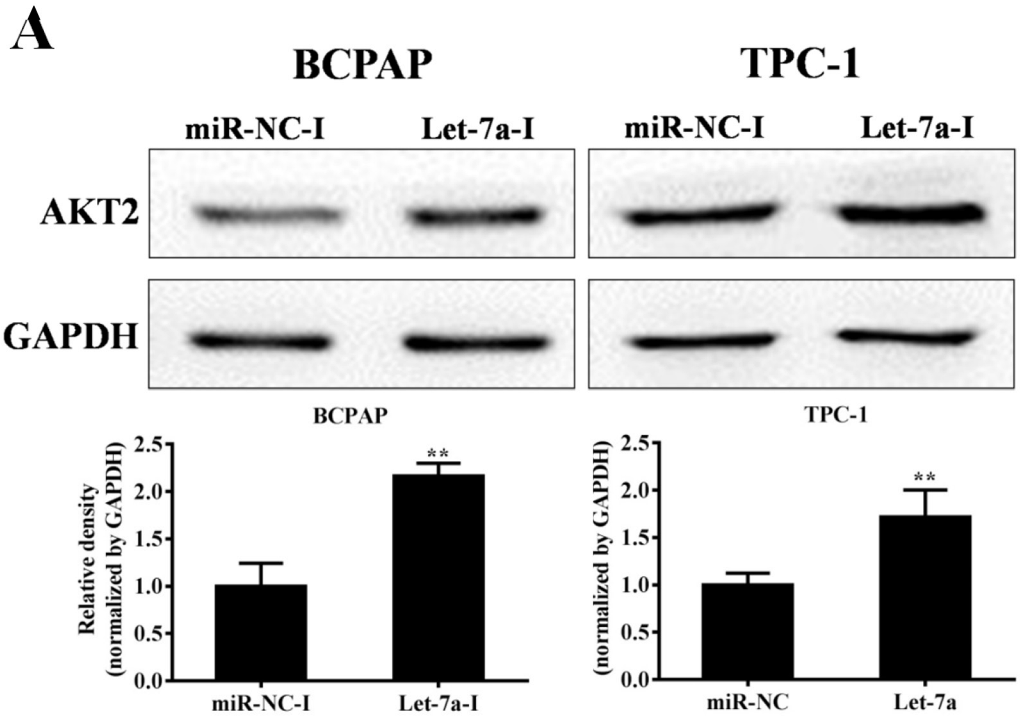

Supplementary Figure 2: AKT2 was up-regulated by let-7a inhibitor. BCPAP and TPC-1 cells were transfected with let-7a inhibitor (let-7a-I) or negative control (miR-NC-I). **(A)** Total proteins were subjected to western blotting and detected for AKT2 expression levels. The density of AKT2 expression levels were quantified and normalized to the level of GAPDH. Error bars represent the mean±SD of triplicate experiments. (**) Significant difference when compared with the miR-NC-I group (P< 0.01).
